# Intra-abdominal bleeding with hemorrhagic shock: a case of adrenal myelolipoma and review of literature

**DOI:** 10.1186/s12893-017-0270-6

**Published:** 2017-06-26

**Authors:** Hui-Pu Liu, Wen-Yen Chang, Shan-Tao Chien, Chin-Wen Hsu, Yu-Chiuan Wu, Wen-Ching Kung, Chun-Min Su, Ping-Hung Liu

**Affiliations:** Department of General Surgery, Kaohsiung Armed Forces General Hospital, No.2, Zhongzheng 1st Rd., Lingya Dist, Kaohsiung City, 802 Taiwan

**Keywords:** Adrenal myelolipoma, Hemorrhagic shock, Fat-content mass, Retroperitoneum

## Abstract

**Background:**

Adrenal myelolipoma is an uncommon, benign, and hormonally non-functioning tumor that is composed of mature adipose tissue and normal hematopoietic tissue. Most cases to date are asymptomatic or have epigastric pain. Acute hemorrhage is the most dramatic manifestation of adrenal myelolipoma; though, it is a rare entity. Hemorrhagic shock due to adrenal myelolipoma, to our knowledge, was much less mentioned so far. Persistent bleeding and uncontrollable hypotension are considered to be absolute indications for immediate surgical operation.

**Case presentation:**

Herein we presented a 32-year-old male patient with initial symptoms of nausea, vomiting, and epigastric pain progressing to altered consciousness and hypotension during ER course. Hemorrhagic shock due to a giant adrenal myelolipoma, R’t was diagnosed. Emergent exploratory laparotomy was executed, and en bloc excision of tumor was done.

**Conclusion:**

Adrenal myelolipoma might be diagnosed as a adjunction to other main causes of illness; furthermore, adrenal myelolipoma could be asymptomatic in lifetime. In our case, however, manifesting as hemorrhage shock was challenging to diagnose step by step; instead, maintaining vital organs perfusion and identifying bleeding sources were to be done. Management of myelolipoma should be done on a case-to-case basis.

## Background

Myelolipoma is a uncommon benign tumor of mesenchymal origin which consists of mature adipose tissue and normal hematopoietic cells. It occurs most often in adrenal gland [1] while extraadrenal (most often in the retroperitoneum) myelolipomas are also reported [2]. To date, most cases are asymptomatic or have epigastric pain. Acute hemorrhage is the most dramatic manifestation of adrenal myelolipoma; though, it is a rare entity. Hemorrhagic shock due to adrenal myelolipoma, to our knowledge, was far less mentioned so far. Myelolipoma accompanied by acute hemorrhage and calcification can complicate making the image-based diagnosis. Computed tomography frequently demonstrates large amounts of fat with areas of interspersed higher-attenuation tissue [3]. It is difficult to differentiate giant adrenal or extraadrenal myelolipomas from other fat-containing soft-tissue masses such as lipoma, liposarcoma, myolipoma, teratoma and an exophytic angioliposarcoma of the kidney [4].

## Case presentation

**Fig. 1:**
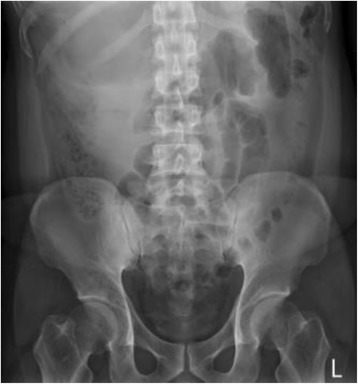
KUB showed a hypodense lesion over right upper quodrant of abdomen

A 32-year-old male patient has history of peptic ulcer disease under medication for a few months, and he presented to emergency department with 1-day duration of nausea, vomiting, and epigastric pain. Abdomen in ovoid shape, tenderness over RUQ of abdomen without rebounding pain, and normoactive bowel sound were found in physical exam. ECG showed sinus tachycardia. Initially, intravenous antacids was administered under tentative diagnosis of exacerbation of peptic ulcer disease; whereas, such illness persisted. On further inquiry, the abdominal pain was irrelevant to trauma, body posture, or food intake. Laboratory data were normal except leukocytosis with white blood cell count of 19,100/uL and neutrophil predominance: 91.2%. KUB showed a huge hypodense lesion over right upper region (Fig. 1).

**Fig. 2:**
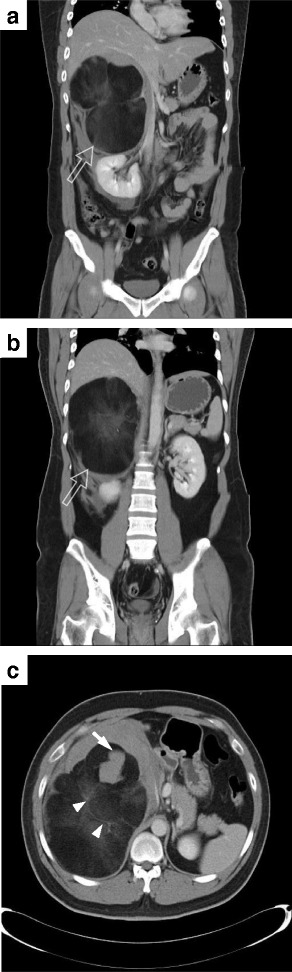
CT of abdomen with contrast showed a huge retroperitoneal majorly fat-content mass (*open arrow*) over right suprarenal (**a** & **b**) with ruptured hemorrhage (*arrow*), and interspersed with enhancing soft tissue components, as smoky appearance (*arrowhead*) (**c**)

Sudden onset of altered consciousness with downhill of blood pressure was noted when awaiting the exam of CT(computerized tomography) of abdomen. He was resuscitated with intravenous fluid hydration and blood transfusion then. And CT of abdomen revealed a huge retroperitoneal majorly fat-content mass with ruptured hemorrhage (Fig. 2).

**Fig. 3:**
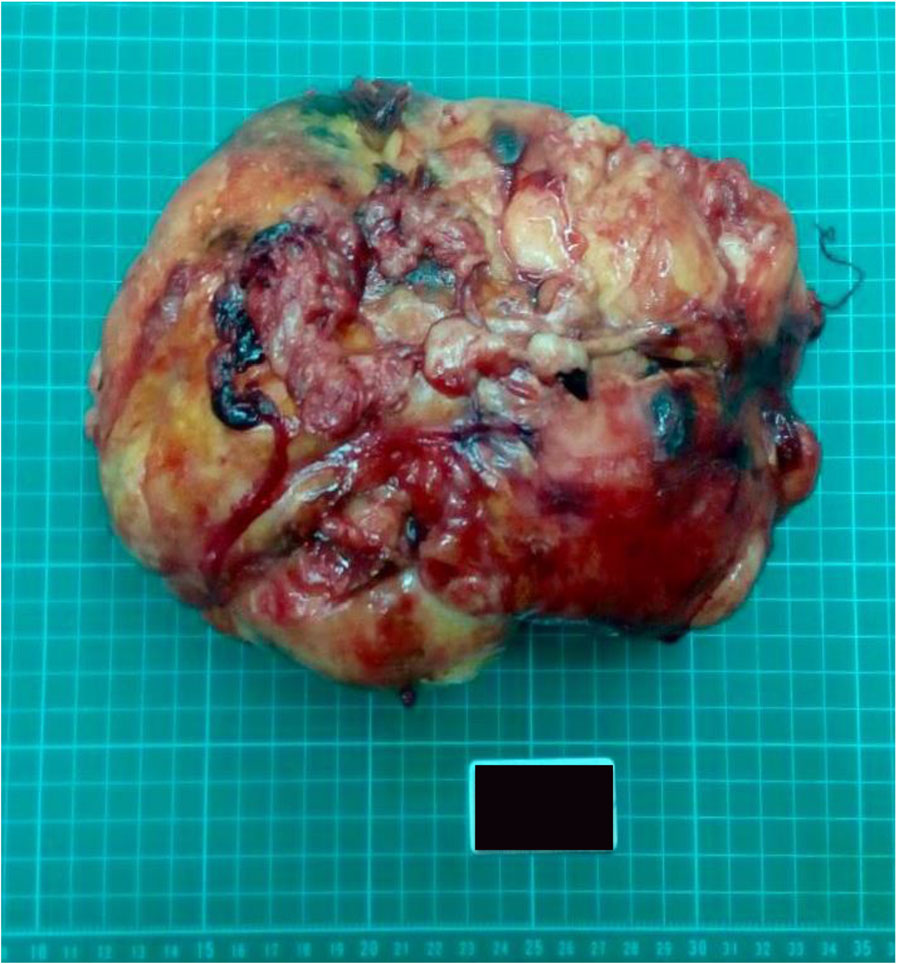
Photograph of gross pathologic specimen shows encapsulated soft and yellow solid mass, measuring 22x16x18 cm^3^, weighed 1450 g

**Fig. 4:**
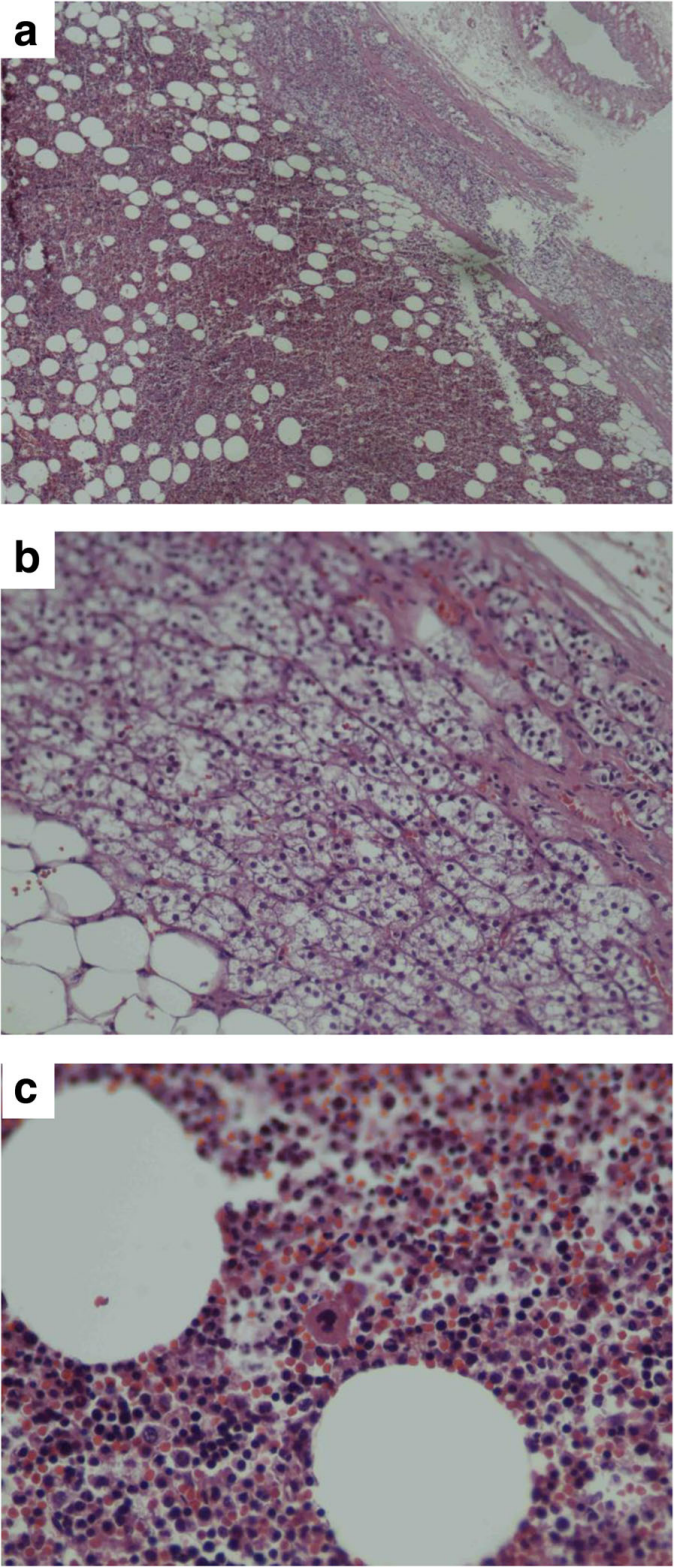
Microphotograph (**a**) (Hematoxylin & eosin, ×100) showed adrenal gland with intraparenchymal myelolipoma. **b **(Hematoxylin & eosin, ×200) showed zone fasciculata of adrenal gland, and zone reticulata was occupied by adipocytes. Numerous myeloid cells and immature red blood cell along with some megakaryocytes can be seen in (**c**) (Hematoxylin & eosin, ×400)

General surgery specialist performed emergent exploratory laparotomy immediately. En bloc excision of tumor was done. During operation, blood clots in abdominal cavity up to 1100 ml, and a huge retroperitoneal tumor with active bleeding, sized about 22x16x18 cm^3^ were found. Total blood loss was 3500 ml. The tumor weighed 1450 g (Fig. 3). Pathology microphotograph revealed numerous mature adipocytes surrounded by myeloid cells in adrenal gland indicating myelolipoma (Fig. 4) The patient led an uneventful postoperative hospital course.

## Discussion

Adrenal myelolipoma was first introduced in 1905 by Gierke, and following given the name myelolipoma by Oberling. It consists of lipoid and hematopoietic elements, and the reticulum is present only in the fatty areas [5]. The incidence of myelolipoma is 0.2% in the general population in reported autopsy studies. Acute hemorrhage is the most significant complication especially in large myelolipomas, and it can be manifested with local pain in the back, epigastrium, or flanks, accompanied with nausea, vomiting, hypotension and anemia [2].

It must be distinguished from extramedullary hematopoietic tumors, which are as below: 1.) more often multiple than solitary, 2.)frequently associated with splenomegaly and hepatomegaly, 3.)secondary to severe anemia (thalassemia, hereditary spherocytosis), various myeloproliferative diseases, myelosclerosis, and skeletal disorders [6].

In image study, computed tomography usually shows well-marginated, heterogeneous masses with macroscopic fat. Interestingly, a smoky appearance with attenuation values around 20-30HU could be found in some area of myelolipoma due to admixture of adipose and hematopoietic cells [2]. If non-invasive studies could not yield definite diagnosis, fine-needle aspiration (FNA) biopsy should be considered [7–9]. Also in cases where expectant management is being considered, FNA can definitively rule out malignancy [9, 10].

Retroperitoneal hemorrhage due to spontaneous rupture of adrenal myelolipoma is very rare, and surgical resection is recommended [11–13]. Persistent bleeding with symptoms of hemorrhagic hypovolemia like refractory hypotension and altered consciousness are considered to be absolute indications for immediate surgical intervention [14]. The CT findings of the lesions with bleeding are not easy to identify. The most important is the difference in size with most bleeding lesions being greater than 10 cm. Sizes have been variably ranging from microscopic lesions to giant masses as big as 31 cm [15].

Though several theories have been proposed, the etiology of myelolipoma remains obscure. Theories include remnants of fetal bone marrow, embolism of bone marrow cells, and hyperplasia of heterotopic reticulum cells [16, 17]. Chang et al. described a case of adrenal myelolipoma with a translocation t(3;21)(q25;p11). A similar change, t(3;21)(q26;p11), is found in hematopoietic neoplasms, such as myelodysplastic syndromes (MDS) and chronic myeloid leukemia (CML) indicating that myelolipoma is a derivative from misplaced hematopoietic cells [18]. Bishop et al. proposed a theory that X-chromosome inactivation in both fat and hematopoietic elements could be the clonal origin of myelolipoma [19]. The relationship between high-energy trauma and the development of adrenal myelolipoma has also been proposed [20], and significant changes in bone marrow hematopoiesis after severe trauma were shown in some studies [21, 22].

Management of myelolipoma should be done on a case-to-case basis. Patients with lesions <10 cm defined as myelolipoma on imaging procedures, should be observed closely for 1–2 years. If patient is asymptomatic and there is no tumor growth then the follow-up can be done at increasing time intervals, however the follow-up will be life-long because interval growth has been reported previously [23, 24].

## Conclusion

For the symptoms and signs of adrenal myelolipoma are nonspecific, it might be diagnosed as a adjunction to other main causes of illness; furthermore, adrenal myelolipoma could be asymptomatic in lifetime. In our case, however, manifesting as hemorrhage shock was challenging to diagnose step by step; instead, maintaining vital organ perfusion and identifying bleeding sources were top priorities. Management of myelolipoma should be done on a case-to-case basis.

## Abbreviations

CT: Computerized tomography
ECG: Electrocardiogram
FNA: Fine-needle aspiration
IV: Intravenous
KUB: Kidney, Ureter, Bladder
RUQ: Right upper quadrant
